# External validation of multidimensional prognostic indices (ADO, BODEx and DOSE) in a primary care international cohort (PROEPOC/COPD cohort)

**DOI:** 10.1186/s12890-016-0305-2

**Published:** 2016-11-11

**Authors:** Maite Espantoso-Romero, Miguel Román Rodríguez, Ana Duarte-Pérez, Jaime Gonzálvez-Rey, Pedro A. Callejas-Cabanillas, Durdica Kasuba Lazic, Berta Anta-Agudo, Pere Torán Monserrat, Rosa Magallon-Botaya, Biljana Gerasimovska Kitanovska, Heidrun Lingner, Radost S. Assenova, Claudia Iftode, Francisco Gude-Sampedro, Ana Clavería, On behalf of the PROEPOC/COPD study group

**Affiliations:** 1 Teis Health Centre, Xerencia de Xestión Integrada, Vigo, Spain; 2 Son Pisà Health Centre, IB-Salut Balears, Palma de Mallorca, Spain; 3 Cangas Health Centre, Xerencia de Xestión Integrada, Vigo, Spain; 4 Matamá Health Centre, Xerencia de Xestión Integrada, Vigo, Spain; 5 Department of Family Medicine, School of Medicine, University of Zagreb, Zagreb, Croatia; 6 Barrio La Salud Health Centre, Santa Cruz de Tenerife, Spain; 7 Family Medicine, Jordi Gol Institute for Primary Care Research, Santa Coloma de Gramenet, Spain; 8 Arrabal Health Centre, Zaragoza, Spain; 9 Preventative Activities and Health Promotion Network (REDIAPP), Instituto Aragonés de Ciencias de la Salud (IACS), Zaragoza, Spain; 10 Department of Family Medicine, University of Sts. Cyril and Methodius, Skopje, Macedonia; 11 Hannover Medical School, Centre for Public Health and Healthcare, Hannover, Germany; 12 Department General Practice, Medical University of Plovdiv, Plovdiv, Bulgaria; 13 Cabinet Medical De Medicina Familiei, Timisoara, Romania; 14 Epidemiology Department, Clinical University Hospital of Santiago de Compostela, Santiago de Compostela, Spain; 15 Preventative Activities and Health Promotion Network (REDIAPP), Santiago de Compostela, Spain; 16 Primary Care, Instituto Investigación Sanitaria Galicia Sur, Xerencia de Xestión Integrada, Vigo, Spain; 17 Preventative Activities and Health Promotion Network (REDIAPP), Vigo, Spain

**Keywords:** Chronic obstructive pulmonary disease, Validation studies as topic, Primary health care, Prognosis

## Abstract

**Background:**

Due to the heterogeneous and systemic nature of the chronic obstructive pulmonary disease (COPD), the new guidelines are oriented toward individualized attention. Multidimensional scales could facilitate its proper clinical and prognostic assessment, but not all of them were validated in an international primary care cohort, different from the original ones used for model development. Therefore, our main aim is to assess the prognostic capacity of the ADO, BODEx and DOSE indices in primary care for predicting mortality in COPD patients and to validate the models obtained in subgroups of patients, classified by revised Global Initiative for Chronic Obstructive Lung Disease (2011) and updated Spanish Guideline (2014). Besides, we want to confirm that the prognostic capacity of all indices increases if the number of exacerbations is substituted by the interval between them and to assess the impact on health of the patient’s lifestyle, social network and adherence to treatment.

**Methods:**

Design: External validation of scales, open and prospective cohort study in primary care.

Setting: 36 health centres in 6 European high, medium and low income countries.

Subjects: 477 patients diagnosed with COPD, captured in clinical visit by their General Practitioner/Nurse.

Predictors: Detailed patient history, exacerbations, lung function test and questionnaires at baseline.

Outcomes: Exacerbations, all-cause mortality and specific mortality, within 5 years of recruitment.

Analysis: Multivariate logistic regression and Cox regression will be used. Possible non-linear effect of the indices will be studied by using Structured Additive Regression models with penalised splines. Subsequently, we will assess different aspects of the regression models: discrimination, calibration and diagnostic precision. Clinical variables modulated in primary care and the interval between exacerbations will be considered and incorporated into the analysis.

**Discussion:**

The Research Agenda for General Practice/Family Medicine highlights that the evidence on predictive values of prognostic indices in primary care is scarce. A prospective cohort like that of *PROEPOC/COPD* provides good opportunities for research into COPD and make communication easier between family practitioners, nursing staff, pneumologists and other professionals, supporting a multi-disciplinary approach to the treatment of these patients.

**Trial registration:**

ISRCTN52402811. Date: 15/01/2015. Prospectively registered.

**Electronic supplementary material:**

The online version of this article (doi:10.1186/s12890-016-0305-2) contains supplementary material, which is available to authorized users.

## Background

### Clinical and prognostic assessment of chronic obstructive pulmonary disease

Chronic Obstructive Pulmonary Disease (COPD) is a major public health problem, as there is considerable underdiagnosis and a high morbimortality. It is the fourth cause of death among males in European countries and its prevalence is expected to continue to increase [1]. Available epidemiological data show a wide variability in the incidence, prevalence and mortality in different countries [2]. In Spain, the EPI-SCAN study [3] found an overall prevalence for COPD of 10.2 % (CI 95 %: 9.2–11.1 %), higher among men than women (15.1 %/5.6 %), with 4.4 % (CI 95 %: 3.8–5.1 %) of cases classified as moderate to very severe (Stages II-IV of the Global Initiative for Chronic Obstructive Lung Disease [GOLD] classification).

COPD is characterized by a chronic poorly reversible airflow limitation, which is usually progressive if appropriate measures are not taken. Although the value for maximum forced expiratory volume in the first second (FEV1) has been the most commonly used prognostic variable, there is increasingly more evidence that FEV1 does not express the complexity and heterogeneity of COPD. Exercise tolerance, exacerbations, comorbidity, social support and quality of life are all dimensions that have been shown to be important predictors of mortality, each with its own strengths and limitations [4]. This has not led to abandoning the use of FEV1, with different cut-off thresholds to the classic ones, as a simple element with a high predictive value [5].

In its 2011 revised version, the GOLD [6] proposed a combined assessment of COPD based on the patient’s level of symptoms (measured on the modified Medical Research Council [MRC] dyspnoea scale or the COPD Assessment Test [CAT] questionnaire) and on the frequency of exacerbations in the last year or the degree of intensity of the obstruction (FEV1). This assessment has established the basis for drawing up guidelines for the individualized therapeutic management of COPD patients. Even though an advance, it caused controversy: depending on the criteria used for the classification, the allocation of patients could vary [7]; and the prognostic capacity of each group has also varied in different studies [8, 9].

In 2012, the *Guía Española de la EPOC (GesEPOC)* [the Spanish COPD guidelines, hereafter *GesEPOC*] became available [10]. These clinical practice guidelines classify COPD into 4 phenotypes: non-exacerbator, mixed COPD-asthma, exacerbator with emphysema and exacerbator with chronic bronchitis. It also links the phenotypes to the therapeutic activity, whether pharmacological or not, but there is still no quantitative evidence regarding the relationship between prognostic variables and each phenotype.

Therefore, the heterogeneous and systematic nature of COPD has meant that, in recent years, numerous variables are considered for its correct clinical and prognostic assessment. This has led to the construction of different multi-component or multi-dimensional scales. In a recent systematic review, 15 indices were analysed [11], most had a moderate to good prognostic value and some gave an appropriate assessment. The BODE index [12] (B = Body mass index; O = airflow Obstruction; D = Dyspnoea*;* and *E* = Exercise capacity, the 6-minute walk test [6MWT]) has been the most widely used, even though the inclusion of the 6MWT and the difficulty of incorporating this aspect for certain groups of patients limits its use in primary care settings.

For use in Primary Care (PC), the requirements for an index are [13]: a) it is simple to record and calculate; b) its components should be easy to record and register; c) its components should individually be clinically significant; d) it should be able to predict severity, with quality of life and mortality as outcomes; and e) it should predict future exacerbations and consumption of resources. With these criteria, the following indices could be selected for use in PC:

1. The ADO (Age, Dyspnoea, Obstruction) index, derived from the BODE index in which age is included and with a higher predictive value than the original BODE; i.e., able to predict the probability of a specific patient dying in the next 3 years [14]. Recently, this scale has been recalibrated [15] in a very large cohort.
2. The BODEx index, a simplified version of BODE in which the 6MWT is eliminated, substituting it with exacerbations without losing predictive capacity [16]. In the updated *GesEPOC* of 2014 [17], its use is proposed as a guide for referring patients and for selecting pharmacological treatment. This index is well known in Spain, but to date has had only limited use in the rest of Europe.
3. The DOSE (Dyspnoea, Obstruction, Smoking, Exacerbation) index is the only one initially designed in PC, and although it first focused on health outcomes, its prognostic capacity has been demonstrated for mortality [18, 19].

The ADO and BODEx indices were designed for large cohorts in a hospital setting, in some cases with outpatients. To be applied in a different care setting, their validation is recommended, as has already been partially done in some studies [20, 21]. For the designers of each index, each one is applicable in primary care and each one has the best prognostic capacity. Comparing published studies, the BODEx [16] has an area under the curve (AUC) of 0.75, which has been exceeded by the modified ADO index [15] with an AUC of 0.80, while the value for the AUC for DOSE was not published [18].

COPD is a preventable and treatable disease, which requires a comprehensive approach in its assessment as well as multi-professional care. In addition to the indices described above, other aspects will be considered that are related to the health outcomes of the patient and to the preventative and/or therapeutic actions [22] usually managed by the patient and by primary care professionals. Such aspects have repeatedly shown that the quality of life of a patient can improve even without changes in functional parameters: a) Smoking and alcohol, with standardized tools in research and clinical practice for measurement and educational intervention. b) Physical exercise: The International Physical Activity Questionnaire (IPAQ) is the only one validated in Spanish and other languages, allowing international comparisons to be made. Nevertheless, there are other measurements that can be used, which have demonstrated the effect of exercise on health outcomes [23]. c) Social aspects, such as social network and family support [24]. d) Pharmacological follow-up: There is an electronic prescription-dispensing system within the electronic medical record (EMR) that allows an assessment of the follow-up of clinical guidelines and a comparison of the drugs prescribed and those collected from the pharmacy by the patient, as a measurement of adherence, essential for control of the disease by the patients themselves [25]. e) Comorbidities: Whether they have an underlying pathophysiological basis or are coincidental in time, comorbidities modulate and/or complicate the evolution of the diseases. Therefore, we will analyse the generic Charlson Comorbidity Index [26] and the specific COMorbidities in Chronic Obstructive Lung Disease (COMCOLD) index, which reflects the combined impact of five important comorbidities from the perspective of the patients and complements existing comorbidity indices that predict mortality [27].

The existence of the EMR makes it possible to incorporate in the follow-up three key aspects that are novel in prognostic studies: a) the interval of time between exacerbations (and not only their number); b) the existence of exacerbations treated in an emergency primary care setting (which could involve treatment of the acute phase, but maintains the medication prior to patient discharge, at present not counted as exacerbation); and c) assessment of adherence.

To sum up, the ADO and BODEx indices validated predominantly in cohorts from hospital settings can have a different prognostic capacity in a PC setting, due to the differences in severity and other clinical and social characteristics that depend on the setting in which patients are treated. Therefore, we need to verify if the capacity of these indices to discriminate and calibrate patients is maintained when they are applied in primary care.

Besides, the ADO, BODEx and DOSE indices, obtained in the PC setting, can show a different prognostic capacity in each subgroup, according to the GOLD classification and for each phenotype according to the *GesEPOC* classification. We expect that the prognostic capacity is better in groups C and D of the GOLD classification and in the exacerbator phenotypes of the *GesEPOC*, because these patients may be more widely represented in the population treated in the hospital and have lower intragroup variability. The prognostic capacity for the GOLD subgroups will vary according to whether CAT or MRC is used for their classification.

The aim of our study is to assess the prognostic capacity of the ADO, BODEx and DOSE indices in primary care, and to validate the models obtained in subgroups of patients, classified by the new combined assessment proposed in the revised GOLD methodology of 2011 and according to the phenotypes proposed in the updated *GesEPOC* of 2014. Besides, we want to confirm that the prognostic capacity of all indices increases if the number of exacerbations is substituted by the interval between them and to assess the impact on health of the patient’s lifestyle, social network and adherence to treatment.

## Method/design

### Design

Open, prospective and multi-centre validating and updating scales study. Patient recruitment started in February 2015 and will last until Dec 31th, 2016. The patients will be followed for 5 years since their recruitment.

Standards of Reporting adhere to the SPIRIT 2013 Statement for protocols.

### Setting

Primary Care Centres, from Vigo Health Authority (Galicia, Spain) and from Balearic Islands Health Authority (Balearic Islands, Spain). Through these centres, doctors/nurses potentially willing to participate were identified during national and international scientific meetings, both in Spain (Aragón, Canarias, Cataluña) and other European countries (Croatia, Bulgaria, Macedonia, Germany and Romania). Subsequently, General Practitioners (GPs) and/or Nurses were invited and informed by e-mail about the description of the background and objectives of the study, as well as the implications of their participation. There are 36 health centres involved.

Study sites can be found in authors affiliations.

### Subjects

Patients diagnosed with COPD. The eligibility criteria for those studied were similar to those used in the original derivation of the models.

#### Inclusion criteria

- patients with a moderate-severe obstruction, defined by a FEV1 lower than 80 % of the expected post-bronchodilator FEV in the presence of a FEV1 quotient/forced vital capacity (FVC) lower than 70 %, diagnosed with COPD
- patients in a stable phase of COPD, clinically defined as at least 6 weeks since the last exacerbation
- acceptance to participate in the study by providing written informed consent.

#### Exclusion criteria

- patients diagnosed with cystic fibrosis, or bronchiectasis that is clinically significant or of a different origin to COPD (tuberculosis, childhood infections, immunodeficiencies, and severe cognitive or mobility impairment)
- patients diagnosed with a severe chronic disease, besides COPD (active malignancy, AIDS, heart failure, severe kidney or liver failure, etc.).

#### Sample size

A validation study has a specific goal: quantifying the performance of an existing model in other data. Sample size requirements for validation studies are not well understood, and there is a dearth of empirical evidence to guide investigators. In some cases it is possible to choose sample size on statistical grounds. We will focus on the precision and accuracy of the performance measures in the new data.

To analyse the ROC curve for each index and the corresponding AUC, the size of the sample was calculated using pROC package, free software available at https://cran.r-project.org/. The formula used was: *power.roc.test (auc = 0.75, sig.level = 0.05, power = 0.9, kappa = 8).*

The selected parameters considered were: a) mortality estimated in 3 years to be 12.5 % [14], which is a proportion of 1 exitus for every eight patients; b) 30 % of the patients belong to subgroups A, C and D of the GOLD classification [9], although there are discrepancies in this proportion according to the study; c) the lowest AUC described for the different indices is 0.75 [15, 17]; and d) Type I error has been set at 5 % and the power of the study at 90 %.

Under these assumptions, 477 patients need to be recruited, of which 30 % should belong to groups A, C and D, with the number of patients in group A = number in group B = number in group C = 133 (15 deaths and 118 living after 3 years). Moreover, 10 % are added for patients expected to be in group B and a further 10 % for losses to the study. This figure will also be adequate for the total number of patients, but not for those in group B. Currently, there is no published prevalence for the *GesEPOC* 2014 phenotypes, therefore, these subgroups cannot be estimated.

### Patient recruitment

The healthcare professionals identified potentially eligible cases, within their quota of assigned patients, through electronic medical records review (10 per professional). Those patients who are diagnosed with COPD will be included, provided that they fulfil the inclusion criteria as explained previously. After obtaining the informed consent, patients will be incorporated in the study if they have already been correctly diagnosed with COPD. The results of a test taken in the last 6 months during a stable phase will be considered valid; if the last test was more than 6 months ago, a new spirometry test will have to be taken. The study personnel will assess whether the patients have COPD stage II–IV using portable and hand-held spirometry. For our purpose, we will determine postbronchodilator FEV1 and Forced Vital Capacity as a measure for airflow obstruction. Ten minutes before measuring lung function, patients will inhale two puffs of 100 μg salbutamol through a spacer. Once a diagnosis is established, the severity of the obstruction is classified in relation to the post-bronchodilator FEV1 value, expressed as a percentage of the expected level.

The undertaking of the spirometry test and its interpretation will be the responsibility of specially trained staff, who has taken an accredited and tutored theoretical and practical course.

### Outcome variables

Survival (time to event) without exacerbation will be assessed during 5 years since patient recruitment. In addition, mortality (time to event) from all causes and those specifically due to respiratory causes will be analysed as health outcome at the end of the study. Data source will be EMR and death registry data if needed. Blind assessment of the outcome to be predicted is not feasible.

Exacerbations are defined as any sustained increase in respiratory symptoms (dyspnoea, cough and/or expectoration) that require modification of the usual treatment and/or hospital care -visit to the Emergency Room or admission- (for the BODEx index), as well as the exacerbations identified at the PC emergencies department (but without changing subsequent treatment). In this definition, admissions for pneumonia, pulmonary embolism and other causes of complications in COPD are not included.

### Predictors

Assessment of predictors is done by GPs/nurses at baseline, without knowledge of the participant’s outcome, following the indices (ADO, BODEx, DOSE) definition. Formulas to calculate them are available in Additional file 1: Table S1.

They will be recorded in a specific database along with detailed patient history: date of birth, sex, height, weight, and body mass index. The spirometry test will be carried out according to protocol recommendations and the date of the first spirometric diagnosis of COPD will be recorded. Dyspnoea will be assessed according to the scale proposed by the British Medical Research Council for the ADO and BODEx indices. Associated diseases will be evaluated using the Charlson Comorbidity Index and COMCOLD, and the presence of other relevant illnesses, not included in Charlson, will be recorded, such as myopathy, osteoporosis, obstructive sleep apnoea-hypopnoea syndrome, and anxiety and depression disorders. The COPD Assessment Test (CAT) questionnaire will be used to assess the impact of COPD on the daily life and welfare of the patient, and its results will be used for categorisation according to the revised 2011 GOLD classification. Participating patients will be asked about tobacco consumption (packets/year), alcohol consumption (yes/no, units/week), physical exercise (Physical Activity Questionnaire short form (IPAQ), daily physical activity intense/moderate/low), current respiratory pharmacological treatment (in the stable phase), influenza and pneumococcal vaccine status, having received advice about their lifestyle and evaluation of the social support of the patient. The patient will be classified in accordance with the phenotypes defined by the *GesEPOC* [17].

The number of exacerbations in the previous years will be counted, in accordance with each index definition.

The ADO and modified ADO (from 0 to 14) indices, the BODEx index, DOSE index, Charlson index, COMCOLD index and the combined assessment of the GOLD score using the four possible models will be automatically calculated when introducing the variables that make up the computerised database.

For clinical monitoring of the patient, but not included in the prognosis model, a laboratory analysis must be carried out and should include: haemogram, determination of alpha-1-antitrypsin levels (if they have never been determined), IgE and eosinophilia in sputum, as minor criteria of GesEPOC. Moreover, if it is an initial diagnosis, a chest radiograph will be requested. An electrocardiogram will be required if pulmonary hypertension or Stages III or IV of the GOLD classification are suspected [6].

### Independent variables in the follow-up

The follow-up will be undertaken by clinical visits every 6 months. When the patient cannot make the appointment, follow-up will be by telephone interview or a review of the EMR. Information about the date of clinical exacerbations, admissions, visits to the emergency department (primary care or hospital) and mortality will be obtained by a review of the patient’s medical record, contact with the patient’s family and/or passive follow-up (from hospital and mortality records). Survival will be evaluated after 5 years. If cases are referred to a second care level, any specialist care appointments will be recorded.

Adverse events and dropouts during follow-up will be recorded, with the reason for them.

### Participant time line

At enrolment, a clinical assessment is performed for all the patients by appropriately trained study personnel. Participants are asked to perform spirometry and complete the study questionnaires (Table 1). Participants are also requested a laboratory analysis and chest radiograph if needed following the GOLD guideline. Every 6 months, their exacerbations will be count up.

**Table 1:** Participants time line

	2014	2015	2016	2015–2021
Planning				
WHAT				
Design	x			
Galician Ethics Committee approval	x			
Other nodes Ethics Committees	x	x		
Funding	x	x		
Web-page design and pilot study	x			
WHO (nodes)				
Galicia	x			
Balearic Islands	x			
Recruitment				
WHAT				
Spirometry		x		
Clinical assesment		x		
MRC		x		
CAT		x		
IPAQ		x		
Laboratory analysis (optional)		x		
Chest radiography (optional)		x		
WHO (nodes)				
Galicia		x		
Balearic Islands		x		
Aragon		x		
Canary Islands		x		
Catalonia		x		
Bulgaria			x	
Croatia		x		
Germany			x	
Macedonia			x	
Romania			x	
FOLLOW-UP				
WHAT				
Exacerbations (type and number)		x	x	x
Mortality and morbidity outcome		x	x	x

In terms of the level of additional risk posed by diagnostic/monitoring procedures as compared to normal clinical practice, these will be performed in accordance with the terms of the treatment guidelines [1] in any Member State concerned.

### Data analysis

The analyst will identify incomplete data and communicate it to the relevant professional for the particular patient. Appropriate test and statistical treatment (multiple imputations) of lost values will be performed.

To assess the discriminatory capacity of the different indices studied (ADO, BODEx and DOSE), logistic regression models and Cox proportional hazard models will be constructed. Initially, the possible non-linear effect of the indices will be studied by using Structured Additive Regression (STAR) models with penalised splines. Subsequently, we will assess different aspects of the regression models: discrimination, calibration and diagnostic precision. For calibration, the Brier score will be used, together with the Hosmer–Lemeshow test for goodness of fit and graphs of the non-parametric estimates of the association between the expected frequencies and those found for the model. To determine the discrimination of the models, ROC curves (and the corresponding AUC) will be calculated. For survival models, the time-dependent ROC will be estimated, as well as its graphical representation. Diagnostic precision will be calculated as the proportion of patients classified incorrectly. For this purpose, the classification rule shall be as follows: each patient will be classified as belonging to the group for which the predicted probability is higher. To correct against optimism, bootstrap techniques will be used.

Statistical analyses will be carried out using BayesX software for the Structured Additive Regression (STAR) models, freely accessible at http://www.statistik.lmu.de/~bayesx/bayesx.html the package*s rms, survival, surv2sampleComp, pROC, risksetROC* of software R [28, 29], available free at http://cran.r-project.org.

## Discussion

COPD in Europe affects between 4 and 10 % of adults and causes between 200,000 and 300,000 deaths annually. Moreover, associated healthcare costs are estimated at around 10,000 million Euros. The European Platform of Public Health Organizations (EPHA) recommended that COPD research should be included among the priority objectives included in Horizon 2020. Moreover, strategy B3 of the European Innovation Partnership on Active and Healthy Aging prioritised COPD among the chronic diseases requiring research.

The Research Agenda for General Practice/Family Medicine [30] of the European General Practice Research Network (EGPRN) established recommendations both for objectives and methodologies coherent with Family Medicine as defined by World Organization of National Colleges, Academies and Associations of General Practitioners/Family Physicians (WONCA). This agenda has been translated by members of our research team to orientate the research strategy in PC in Spain. The Research Agenda highlights that the evidence on predictive values of all kinds of findings, test or prognostic indices in primary care settings is scarce. Many tests have not been formally evaluated in primary care; different prevalence settings are then used, with more or less selected populations, and often result in unrealistically high prevalence estimates for chronic disease. This is problematic as these results are then used to conclude that GPs are not good at detecting disease and many articles then recommend indices for prognosis in unselected populations or to identify patients to be treated.

In this context, a prospective cohort like that of *PROEPOC/COPD* provides good opportunities for learning about the clinical course of COPD in a PC population, which represents all patients with COPD, from the mildest to the most severe cases. Considering the characteristics of the initial cohort, and the higher survival expected in a primary care setting, ADO and BODEx recalibration may be necessary, as had to be done to combine the cohorts of international studies [15]. The International Primary Care Respiratory Group (IPCRG) set up the Uncovering and Noting Long-term Outcomes in COPD and asthma to enhance Knowledge (UNLOCK) cohort [31]. Kruis [32] evaluated the external validity of 6 large pharmaceutically-sponsored COPD studies (LPCS) and compared them to the characteristics of the patients in 7 database registers that constitute the UNLOCK cohort: between 53 and 84 % of the patients treated in primary care did not fulfil the inclusion criteria of the cohorts recruited in the hospital setting.

In this study, prognostic indices (ADO, BODEx and DOSE) will be assessed for COPD, in different subgroups of patients. Therefore, it will influence the functioning of the health system, because the classification of COPD patients will individualize preventative activities and/or treatments indicated for each patient, modify referral procedures between general family practitioners-nursing staff and specialty-primary care and improve knowledge/self-management of the disease by patients themselves and their families.

The good technical quality of the information obtained is essential for the results of the study to be valid and to reduce variability. Standardization of protocols, including web-page form were sent to the researchers, reviewed and translated in each country by the node leaders. Group reunions are planned during EGPRN meetings, twice per year. The study protocol was presented and reviewed at Barcelona meeting, 11st May, 2014. Later, three reunions were organized (Heraklion, Timisoara and Edirne). The project was presented at UNLOCK board during Singapore IPCRG meeting in 2015. For quality control, the following procedures will be in place throughout the different stages of the study in each node: daily calibration of spirometers; automation of filter variables and forms; computer-based administration of questionnaires; accreditation of the training of the nursing staff in spirometry; updating of clinical knowledge about COPD, data monitoring three times per year. An international workshop about spirometry was organized during WONCA Istanbul meeting in 2015 and web seminars will be scheduled (one per year).

TRIPOD Group [33] encourages complete and transparent reporting reflecting study design and conduct, and even enter the study in registers that accommodate observational studies such as ISRCTN. We try to help readers submitting the TRIPOD reporting template as Additional file 1: Table S2.

The information gathered will allow research into COPD and make communication easier between family practitioners, nursing staff, pneumologists and other professionals, supporting a multi-disciplinary approach to the treatment of these patients. Both GOLD and the phenotypes recently presented in the updated *GesEPOC* have this same orientation. Soriano [34] compared the different groups of combined assessment and the predictive validity of the old and new GOLD methodologies, speculating that there may be a higher percentage of type B COPD in primary care and, therefore, the need for a longer follow-up period. The *GesEPOC* 2014 uses prognostic indices (BODEx, with 5 as the cut-off threshold) to refer patients [17].

The UNLOCK cohort has demonstrated the superiority of DOSE over ADO. However, in a primary care-based cohort, no study in a European setting has included the BODEx index in the comparative analysis. The main aim of the present study, and one of its contributions to this debate, is to undertake a comparative analysis among these indices and with DOSE, in the same patients and with common recruitment criteria. Moreover, the PROEPOC/COPD study gives priority to issues about preventative and/or therapeutic actions usually managed by the patient and by primary care professionals that should be tackled in that setting.

The usefulness of these indices will be determined by their application in primary care [35] and the possibility of personalized treatment, as has been established for a considerable time for cardiovascular diseases. Multi-dimensional scales are tools that should be analysed and used in relation to these guidelines [36].

## Additional file

Additional file 1: Table S1. Calculation of the indices. **Table S2**. TRIPOD Checklist: Prediction Model Validation (DOCX 27 kb)

## Abbreviations

6MWT: 6-minute walk test
ADO: Age, dyspnoea, obstruction index
AUC: Area under the curve
BODE: Body mass index, airflow obstruction, dyspnoea, exercise capacity
BODEx: Body mass index, airflow obstruction, dyspnoea, exacerbations
CAT: COPD assessment test
COMCOLD: COMorbidities in chronic obstructive lung disease
COPD: Chronic obstructive pulmonary disease
DOSE: Dyspnoea, obstruction, smoking, exacerbation
EGPRN: European general practice research network
EMR: Electronic medical record
FEV1: Forced expiratory volume in the first second
GesEPOC: Guía Española de la enfermedad pulmonar obstructiva (Spanish guide for chronic obstructive lung disease)
GOLD: Global initiative for chronic obstructive lung disease
GP: General practitioner
IPAQ: International physical activity questionnaire
IPCRG: International primary care respiratory group
MRC: Medical research council
PC: Primary care
STAR: Structured additive regression
UNLOCK: Uncovering and noting long-term outcomes in COPD and asthma to enhance knowledge
WONCA: World Organization of National Colleges, academies and associations of general practitioners/family medicine
