# Supplementary material for: External validation of multidimensional prognostic indices (ADO, BODEx and DOSE) in a primary care international cohort (PROEPOC/COPD cohort)
Source: BMC Pulm Med. 2016 Nov 11;16:143. doi: 10.1186/s12890-016-0305-2 (PMC5106777; doi:10.1186/s12890-016-0305-2)
Supplement: Additional file 1: Table S1. — Calculation of the indices. Table S2. TRIPOD Checklist: Prediction Model Validation (DOCX 27 kb) [file 12890_2016_305_MOESM1_ESM.docx]

**Additional file**

**Table S1. Calculation of the indices.**

|  | **Allocation of points to the ADO index** | | | | | | | | | | | | |
| --- | --- | --- | --- | --- | --- | --- | --- | --- | --- | --- | --- | --- | --- |
| Points | | 0 | 1 | 2 | 3 | | 4 | | | | 5 | |  |
| FEV1 (%) | | ≥65 | ≥36–64 | ≤35 |  | |  | | | |  | |  |
| Dyspnoea (MRC) | | 0-1 | 2 | 3 | 4 | |  | | | |  | |  |
| Age (years) | | 40-49 | 50-59 | 60-69 | 70-79 | | 80-89 | | | | ≥90 | |  |
|  |  |  |  |  |  | | | |  | |  | |  |
|  |  |  |  |  |  | | | |  | |  | |  |
|  | **Allocation of points to the BODEx index** | | | | | | | | | | | | |
| Points | | 0 | 1 | 2 | 3 | | |  | |  | |  | |
| FEV1 (%) | | ≥65 | 50-64 | 36-49 | <36 | | |  | |  | |  | |
| Dyspnoea (MRC) | | 0-1 | 2 | 3 | 4 | | |  | |  | |  | |
| BMI | | ≥ 21 | <21 |  |  | | |  | |  | |  | |
| Exacerbations | | 0 | 1-2 | ≥ 3 |  | | |  | |  | |  | |
|  |  |  |  |  |  | | |  | |  | |  | |
|  | **Allocation of points to the DOSE index** | | | | | | | | | | | | |
| Points | | 0 | 1 | 2 | 3 | | |  | |  | |  | |
| Dyspnoea (MRC) | | 0-1 | 2 | 3 | 4 | | |  | |  | |  | |
| FEV1 (%) | | ≥50 | 30-49 | <30 |  | | |  | |  | |  | |
| Smoker | | No | Yes |  |  | | |  | |  | |  | |
| Exacerbations (last 6 months) | | 0-1 | 2 | >3 |  | | |  | |  | |  | |
|  |  |  |  |  |  | | |  | |  | |  | |
|  | **GOLD classification** | | | | | | | | | | | | |
|  |  | MRC 0-1 | MRC ≥ 2 |  | |  | | |  |  | |  | |
|  |  |  |  |  | |  | | |  |  | |  | |
|  |  |  |  |  | | | | |  |  | |  | |
| Obstruction | 4=FEV1<30 | C | D | >2 |  | | | |  |  | |  | |
|  | 3=FEV1<50 |  |  |  | Exacerbations | | | | |  | |  | |
|  | 2=FEV1<80 |  |  | 1 |  |  |  |  |  |  | |  | |
|  | 1=FEV1≥80 | A | B |  |  | | | |  |  | |  | |
|  |  |  |  | 0 |  | | | |  |  | |  | |
|  |  |  |  |  |  | | | |  |  | |  | |
|  |  | CAT<10 | CAT ≥10 |  |  | | | |  |  | |  | |
|  |  |  |  |  |  | | | |  |  | |  | |

**Table S2. TRIPOD Checklist: Prediction Model Validation**

| **Section/Topic** | **Item** | **Checklist Item** | **Page** |
| --- | --- | --- | --- |
| **Title and abstract** | | | |
| Title | 1 | Identify the study as developing and/or validating a multivariable prediction model, the target population, and the outcome to be predicted. | 1 |
| Abstract | 2 | Provide a summary of objectives, study design, setting, participants, sample size, predictors, outcome, statistical analysis, results, and conclusions. | 2-4 |
| **Introduction** | | | |
| Background and objectives | 3a | Explain the medical context (including whether diagnostic or prognostic) and rationale for developing or validating the multivariable prediction model, including references to existing models. | 5-9 |
|  | 3b | Specify the objectives, including whether the study describes the development or validation of the model or both. | 9 |
| **Methods** | | | |
| Source of data | 4a | Describe the study design or source of data (e.g., randomized trial, cohort, or registry data), separately for the development and validation data sets, if applicable. | 9 |
|  | 4b | Specify the key study dates, including start of accrual; end of accrual; and, if applicable, end of follow-up. | 9 |
| Participants | 5a | Specify key elements of the study setting (e.g., primary care, secondary care, general population) including number and location of centres. | 9 |
|  | 5b | Describe eligibility criteria for participants. | 11 |
|  | 5c | Give details of treatments received, if relevant. |  |
| Outcome | 6a | Clearly define the outcome that is predicted by the prediction model, including how and when assessed. | 11, 12, 16 |
|  | 6b | Report any actions to blind assessment of the outcome to be predicted. |  |
| Predictors | 7a | Clearly define all predictors used in developing or validating the multivariable prediction model, including how and when they were measured. | 12-15 |
|  | 7b | Report any actions to blind assessment of predictors for the outcome and other predictors. |  |
| Sample size | 8 | Explain how the study size was arrived at. | 10-11 |
| Missing data | 9 | Describe how missing data were handled (e.g., complete-case analysis, single imputation, multiple imputation) with details of any imputation method. | 15 |
| Statistical analysis methods | 10c | For validation, describe how the predictions were calculated. |  |
|  | 10d | Specify all measures used to assess model performance and, if relevant, to compare multiple models. | 15 |
|  | 10e | Describe any model updating (e.g., recalibration) arising from the validation, if done. | 15 |
| Risk groups | 11 | Provide details on how risk groups were created, if done. |  |
| Development vs. validation | 12 | For validation, identify any differences from the development data in setting, eligibility criteria, outcome, and predictors. |  |
| **Results** | | | |
| **Discussion** | | | |

| Limitations | 18 | Discuss any limitations of the study (such as nonrepresentative sample, few events per predictor, missing data). | 11, 15 |
| --- | --- | --- | --- |
| Interpretation | 19a | For validation, discuss the results with reference to performance in the development data, and any other validation data. |  |
|  | 19b | Give an overall interpretation of the results, considering objectives, limitations, results from similar studies, and other relevant evidence. |  |
| Implications | 20 | Discuss the potential clinical use of the model and implications for future research. | 16-17 |

| **Other information** | | | |
| --- | --- | --- | --- |
| Supplementary information | 21 | Provide information about the availability of supplementary resources, such as study protocol, Web calculator, and data sets. | Supp. |
| Funding | 22 | Give the source of funding and the role of the funders for the present study. | 20 |
